# Screening for atrial fibrillation after stroke: is targeted patient selection the key?

**DOI:** 10.1055/s-0044-1791750

**Published:** 2024-11-11

**Authors:** Thaís Leite Secchi, Luciano A. Sposato

**Affiliations:** 1 Hospital Moinhos de Vento, Departamento de Neurologia, Porto Alegre RS, Brazil.; 2 Western University, Schulich School of Medicine and Dentistry, Departments of Clinical Neurological Sciences, Epidemiology and Biostatitistics, and Anatomy and Cell Biology, London, Ontario Canada.; 3 Western University, Heart & Brain Lab, London, Ontario, Canada.

Atrial fibrillation (AF) is a major cause of stroke, accounting for up to one-third of ischemic strokes. 1 Due to its often asymptomatic and paroxysmal nature, AF frequently goes undiagnosed. Consequently, up to 30% of patients initially classified as having cryptogenic stroke are later found to have AF. 2 Strokes related to AF typically result from cardioembolism to large cerebral arteries, leading to more severe outcomes compared with other stroke causes. 1 2 3

Given the proven efficacy of oral anticoagulants (OACs) in preventing recurrent cardioembolic strokes, screening for AF after stroke using ECG monitoring, followed by OAC treatment, has been proposed. 4 However, the current practice of AF screening varies widely across different healthcare settings, primarily due to the lack of standardized guidelines on the optimal duration and method for monitoring. Traditional short-term monitoring, typically limited to 24 hours, may miss paroxysmal AF episodes. While extended ECG monitoring is more effective, it involves higher costs and logistical challenges. A global survey conducted across 61 countries revealed that cardiac monitoring beyond 24 hours was implemented in only 17% of participating hospitals, mainly due to limited technical and human resources. 5

Several risk scores have been developed to predict AF following a stroke, aiming to identify patients at high risk for AF based on their clinical characteristics. 6 However, these scores differ in their variables, outcomes, and ease of use, often requiring additional tests that limit their practicality in routine clinical settings. In previous studies, most of these scores have demonstrated only modest discrimination, despite incorporating several biomarkers. The added complexity and cost further reduce their simplicity and practicality for everyday clinical use. As a result, despite their potential, these tools have not yet been incorporated into clinical guidelines. 7 8

In this issue of *Arquivos de Neuro-Psiquiatria* , Teixeira et al. 9 present a new clinical score tool designed to predict the risk of incident AF during post-stroke follow-up. Based on a retrospective analysis of 872 patients hospitalized for cerebral ischemia between 2014 and 2021 at a tertiary stroke center, this tool aims to enhance screening frequency and risk factor management. Out of 1,025 initially considered patients, 153 were excluded due to prior AF diagnosis or insufficient medical data. The analysis revealed that 9.05% of the 872 ischemic stroke patients developed incident AF during a median follow-up of 12 months.

Markers of atrial disease, such as left atrial enlargement, septal aneurysm, and thrombus in the left atrial appendage, were identified in 20% of the patients and were linked to a higher risk of embolic events, even in the absence of detected AF during the acute phase. The study found that left atrial size ≥42 mm, age ≥70 years, presence of an interatrial septal aneurysm, and NIHSS score ≥6 were significantly correlated with the occurrence of AF. When combined, these factors achieved an area under the curve (AUC) of 0.77. The predictive score, which ranges from 0 to 6, demonstrated that each additional point in the score increased the risk of AF by 2.3 times. Notably, a score of ≥2 points was linked to a 6.7-fold higher risk of developing AF.

The authors correctly acknowledged several limitations related to the retrospective design of the study. Conducted at a single tertiary stroke center, the study's findings may not be broadly generalizable to other populations. Inconsistencies in the frequency of Holter monitor use were noted, varying based on individual clinical judgments. Additionally, the score relies on clinical and echocardiographic variables, which may not encompass the full range of risk factors for AF. Emerging biomarkers, such as natriuretic peptides, and advanced imaging techniques, like cardiac MRI, have shown potential in refining AF risk prediction models. As the authors rightly point out, further studies are needed to externally validate the tool in diverse populations and to compare its predictive performance with existing models. The long-term impact of incorporating this score into clinical practice also requires additional evaluation.

The study's findings highlight the potential for predictive tools to enhance secondary stroke prevention strategies, particularly in resource-limited settings, and address the challenges associated with AF detection. Identifying AF after a stroke is challenging due to the shared risk factors between AF-related and non-AF-related strokes, making it difficult to determine which patients would benefit most from extended monitoring. Future research will be crucial in validating this tool and exploring its broader application in diverse clinical settings. While intensive or prolonged monitoring might increase the identification of subclinical AF, its impact on reducing stroke recurrence remains uncertain.
